# 2,2-Dimethyl-5-[(pyridin-2-yl­amino)­methyl­idene]-1,3-dioxane-4,6-dione

**DOI:** 10.1107/S1600536810053250

**Published:** 2011-01-08

**Authors:** Jian-you Shi, Jin-qi Li, Rong-sheng Tong, He Lin, Chen Lu

**Affiliations:** a Department of Pharmacy, Sichuan Academy of Medical Science and Sichuan Provincial, People’s Hospital, Chengdu 610072, People’s Republic of China

## Abstract

In the title compound, C_12_H_12_N_2_O_4_, the dihedral angle between the pyridine and enamine planes is 3.5 (3)°, while the angle between the dioxanedione (seven atoms) and enamine planes is 4.6 (3)°. The dioxane ring approximates an envelope conformation.

## Related literature

The title compound is an inter­mediate in the synthesis of 4(1*H*)-quinolone-based drugs. For the synthesis and structures of related anti­tumor precursors, see: Cassis *et al.* (1985 ▶); Ruchelman *et al.* (2003 ▶); Shi *et al.* (2009 ▶).
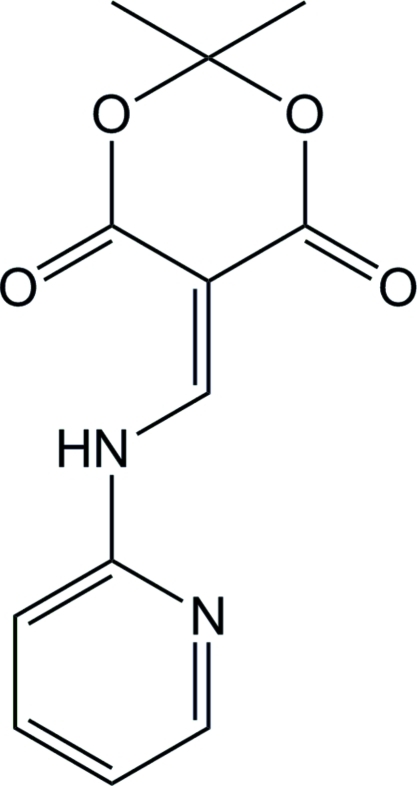


## Experimental

### 

#### Crystal data

C_12_H_12_N_2_O_4_
*M* *_r_* = 248.24
Monoclinic, 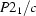

*a* = 8.7344 (10) Å
*b* = 13.9712 (15) Å
*c* = 9.4744 (11) Å
β = 94.601 (11)°
*V* = 1152.4 (2) Å^3^
*Z* = 4
Mo *K*α radiation
μ = 0.11 mm^−1^
*T* = 293 K
0.22 × 0.18 × 0.16 mm

#### Data collection

Oxford Diffraction Xcalibur diffractometer with an Eos CCD detector
Absorption correction: multi-scan (*CrysAlis PRO*; Oxford Diffraction, 2009 ▶) *T*_min_ = 0.997, *T*_max_ = 1.0
4873 measured reflections
2334 independent reflections
1659 reflections with *I* > 2σ(*I*)
*R*_int_ = 0.018

#### Refinement

*R*[*F* ^2^ > 2σ(*F* ^2^)] = 0.040
*wR*(*F* ^2^) = 0.096
*S* = 1.03
2334 reflections
166 parameters
1 restraint
H-atom parameters constrained
Δρ_max_ = 0.14 e Å^−3^
Δρ_min_ = −0.14 e Å^−3^

### 

Data collection: *CrysAlis PRO* (Oxford Diffraction, 2009 ▶); cell refinement: *CrysAlis PRO*; data reduction: *CrysAlis PRO*; program(s) used to solve structure: *SHELXS97* (Sheldrick, 2008 ▶); program(s) used to refine structure: *SHELXL97* (Sheldrick, 2008 ▶); molecular graphics: *SHELXTL* (Sheldrick, 2008 ▶); software used to prepare material for publication: *OLEX2* (Dolomanov *et al.*, 2009 ▶).

## Supplementary Material

Crystal structure: contains datablocks I, global. DOI: 10.1107/S1600536810053250/bh2327sup1.cif

Structure factors: contains datablocks I. DOI: 10.1107/S1600536810053250/bh2327Isup2.hkl

Additional supplementary materials:  crystallographic information; 3D view; checkCIF report

## Figures and Tables

**Fig. 1.**
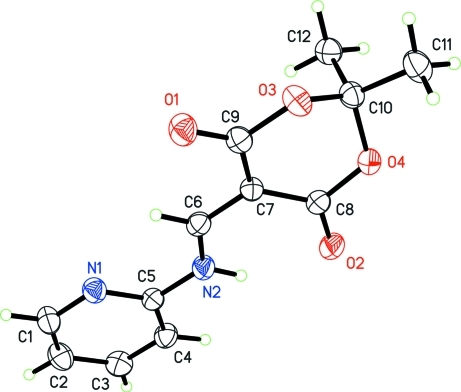
ORTEP-like view of the title compound.

## supplementary crystallographic information

### Comment

The title compound is a key intermediates, which can be used to synthesize 4(1*H*)-quinolone derivatives by thermolysis. These compounds can be used as precursors for anti-malarial and anticancer agents (Cassis *et al.*, 1985; Ruchelman *et al.*, 2003; Shi *et al.*, 2009).

### Experimental

A mixture of 2,2-dimethyl-1,3-dioxane-4,6-dione (1.44 g, 0.01 mol) and methylorthoformate (1.27 g, 0.012 mol) was refluxed for 2.5 h. Then pyridin-2-amine (0.94 g, 0.01 mol) was added and the mixture was refluxed for 4 h, then poured into cold water and filtered, to afford the title compound as a powder. Single crystals were obtained by slow evaporation of a CH_2_Cl_2_-methanol solution over 3 days.

### Refinement

All H atoms were placed in calculated positions, with C—H bond lengths fixed to 0.93 (aromatic CH), 0.96 (methyl CH_3_) or 0.86 Å (NH group). Isotropic displacement parameters for H atoms were calculated as 1.5 (methyl) or 1.2 (other H atoms) times that of the equivalent displacement parameter of the carrier C atom.

### Crystal data

**Table:**

C_12_H_12_N_2_O_4_	*F*(000) = 520
*M**_r_* = 248.24	*D*_x_ = 1.431 Mg m^−^^3^
Monoclinic, *P*2_1_/*c*	Mo *K*α radiation, λ = 0.7107 Å
Hall symbol: -P 2ybc	Cell parameters from 1780 reflections
*a* = 8.7344 (10) Å	θ = 3.1–29.2°
*b* = 13.9712 (15) Å	µ = 0.11 mm^−^^1^
*c* = 9.4744 (11) Å	*T* = 293 K
β = 94.601 (11)°	Block, colourless
*V* = 1152.4 (2) Å^3^	0.22 × 0.18 × 0.16 mm
*Z* = 4	

### Data collection

**Table:**

Oxford Diffraction Xcalibur diffractometer with an Eos CCD detector	2334 independent reflections
Radiation source: fine-focus sealed tube	1659 reflections with *I* > 2σ(*I*)
graphite	*R*_int_ = 0.018
Detector resolution: 16.0874 pixels mm^-1^	θ_max_ = 26.4°, θ_min_ = 3.4°
ω scans	*h* = −10→9
Absorption correction: multi-scan (*CrysAlis PRO*; Oxford Diffraction, 2009)	*k* = −17→16
*T*_min_ = 0.997, *T*_max_ = 1.0	*l* = −11→11
4873 measured reflections	

### Refinement

**Table:**

Refinement on *F*^2^	Secondary atom site location: difference Fourier map
Least-squares matrix: full	Hydrogen site location: inferred from neighbouring sites
*R*[*F*^2^ > 2σ(*F*^2^)] = 0.040	H-atom parameters constrained
*wR*(*F*^2^) = 0.096	*w* = 1/[σ^2^(*F*_o_^2^) + (0.0383*P*)^2^ + 0.0655*P*] where *P* = (*F*_o_^2^ + 2*F*_c_^2^)/3
*S* = 1.03	(Δ/σ)_max_ < 0.001
2334 reflections	Δρ_max_ = 0.14 e Å^−^^3^
166 parameters	Δρ_min_ = −0.14 e Å^−^^3^
1 restraint	Extinction correction: *SHELXL97* (Sheldrick, 2008), Fc^*^=kFc[1+0.001xFc^2^λ^3^/sin(2θ)]^-1/4^
0 constraints	Extinction coefficient: 0.0060 (9)
Primary atom site location: structure-invariant direct methods	

### Fractional atomic coordinates and isotropic or equivalent isotropic displacement parameters (Å^2^)

**Table:**

	*x*	*y*	*z*	*U*_iso_*/*U*_eq_	
O3	0.21039 (13)	0.15916 (7)	0.50800 (11)	0.0497 (3)	
O4	0.37760 (12)	0.11610 (7)	0.33485 (12)	0.0504 (3)	
C6	0.22965 (17)	−0.10004 (11)	0.49185 (16)	0.0421 (4)	
H6	0.1613	−0.1134	0.5596	0.050*	
O1	0.10857 (14)	0.05240 (7)	0.64457 (11)	0.0556 (3)	
O2	0.44968 (12)	−0.03377 (8)	0.30745 (12)	0.0559 (3)	
N2	0.29233 (14)	−0.17386 (8)	0.43038 (13)	0.0455 (3)	
H2	0.3571	−0.1614	0.3692	0.055*	
C7	0.25719 (16)	−0.00541 (10)	0.46383 (15)	0.0388 (3)	
N1	0.17056 (15)	−0.28950 (8)	0.55799 (13)	0.0474 (4)	
C1	0.1423 (2)	−0.38227 (11)	0.58158 (18)	0.0532 (4)	
H1	0.0776	−0.3976	0.6515	0.064*	
C9	0.18429 (18)	0.06634 (10)	0.54492 (16)	0.0428 (4)	
C8	0.36562 (17)	0.02142 (11)	0.36380 (16)	0.0434 (4)	
C5	0.26224 (16)	−0.27096 (10)	0.45637 (16)	0.0400 (4)	
C3	0.2993 (2)	−0.43431 (11)	0.40519 (18)	0.0547 (5)	
H3	0.3432	−0.4829	0.3548	0.066*	
C4	0.32929 (19)	−0.34013 (11)	0.37743 (17)	0.0487 (4)	
H4	0.3930	−0.3233	0.3074	0.058*	
C12	0.11654 (19)	0.16341 (11)	0.26210 (16)	0.0493 (4)	
H12B	0.1452	0.1783	0.1689	0.074*	
H12C	0.0343	0.2048	0.2851	0.074*	
H12A	0.0833	0.0980	0.2649	0.074*	
C11	0.3145 (2)	0.27839 (11)	0.36841 (19)	0.0606 (5)	
H11A	0.3519	0.2923	0.2781	0.091*	
H11C	0.3969	0.2843	0.4412	0.091*	
H11B	0.2342	0.3226	0.3864	0.091*	
C2	0.2036 (2)	−0.45560 (11)	0.50844 (18)	0.0582 (5)	
H2A	0.1806	−0.5189	0.5286	0.070*	
C10	0.25227 (18)	0.17780 (10)	0.36764 (16)	0.0439 (4)	

### Atomic displacement parameters (Å^2^)

**Table:**

	*U*^11^	*U*^22^	*U*^33^	*U*^12^	*U*^13^	*U*^23^
O3	0.0704 (8)	0.0405 (6)	0.0386 (6)	−0.0001 (5)	0.0061 (5)	−0.0014 (5)
O4	0.0432 (7)	0.0437 (6)	0.0656 (8)	−0.0011 (5)	0.0137 (5)	0.0085 (5)
C6	0.0384 (9)	0.0485 (9)	0.0395 (9)	0.0012 (7)	0.0042 (7)	−0.0004 (7)
O1	0.0669 (8)	0.0573 (7)	0.0448 (7)	0.0015 (6)	0.0178 (6)	0.0005 (5)
O2	0.0470 (7)	0.0543 (7)	0.0692 (8)	0.0047 (6)	0.0208 (6)	0.0026 (6)
N2	0.0452 (8)	0.0426 (8)	0.0502 (8)	0.0012 (6)	0.0140 (6)	0.0015 (6)
C7	0.0374 (8)	0.0399 (8)	0.0390 (8)	−0.0006 (7)	0.0034 (6)	0.0022 (7)
N1	0.0516 (9)	0.0447 (8)	0.0471 (8)	0.0022 (6)	0.0102 (6)	0.0016 (6)
C1	0.0620 (11)	0.0477 (10)	0.0511 (10)	−0.0035 (9)	0.0121 (8)	0.0056 (8)
C9	0.0460 (9)	0.0445 (9)	0.0373 (9)	−0.0010 (7)	−0.0001 (7)	0.0006 (7)
C8	0.0372 (9)	0.0438 (9)	0.0491 (9)	−0.0005 (7)	0.0020 (7)	0.0017 (7)
C5	0.0376 (8)	0.0403 (9)	0.0418 (9)	−0.0009 (7)	0.0022 (6)	0.0015 (7)
C3	0.0624 (12)	0.0465 (10)	0.0557 (11)	0.0058 (8)	0.0072 (9)	−0.0100 (8)
C4	0.0478 (10)	0.0521 (10)	0.0477 (10)	0.0015 (8)	0.0123 (8)	−0.0025 (7)
C12	0.0534 (10)	0.0523 (9)	0.0422 (10)	0.0029 (8)	0.0047 (7)	0.0012 (7)
C11	0.0704 (13)	0.0451 (10)	0.0651 (12)	−0.0087 (9)	−0.0009 (9)	0.0058 (8)
C2	0.0728 (13)	0.0414 (9)	0.0606 (12)	−0.0031 (9)	0.0069 (9)	0.0017 (8)
C10	0.0495 (10)	0.0411 (9)	0.0419 (9)	−0.0002 (7)	0.0078 (7)	0.0016 (7)

### Geometric parameters (Å, °)

**Table:**

O3—C9	1.3671 (16)	C1—C2	1.370 (2)
O3—C10	1.4311 (18)	C5—C4	1.381 (2)
O4—C8	1.3567 (17)	C3—H3	0.9300
O4—C10	1.4465 (18)	C3—C4	1.371 (2)
C6—H6	0.9300	C3—C2	1.370 (2)
C6—N2	1.3242 (18)	C4—H4	0.9300
C6—C7	1.374 (2)	C12—H12B	0.9600
O1—C9	1.2112 (17)	C12—H12C	0.9600
O2—C8	1.2172 (17)	C12—H12A	0.9600
N2—H2	0.8600	C12—C10	1.502 (2)
N2—C5	1.4072 (18)	C11—H11A	0.9600
C7—C9	1.442 (2)	C11—H11C	0.9600
C7—C8	1.442 (2)	C11—H11B	0.9600
N1—C1	1.3415 (19)	C11—C10	1.507 (2)
N1—C5	1.3263 (18)	C2—H2A	0.9300
C1—H1	0.9300		
			
O3—C9—C7	115.68 (13)	C5—N2—H2	117.1
O3—C10—O4	110.21 (11)	C5—N1—C1	116.06 (13)
O3—C10—C12	110.33 (13)	C5—C4—H4	120.9
O3—C10—C11	106.51 (12)	C3—C4—C5	118.16 (15)
O4—C8—C7	116.85 (13)	C3—C4—H4	120.9
O4—C10—C12	110.35 (12)	C3—C2—C1	118.99 (15)
O4—C10—C11	106.15 (13)	C3—C2—H2A	120.5
C6—N2—H2	117.1	C4—C5—N2	119.12 (13)
C6—N2—C5	125.74 (13)	C4—C3—H3	120.6
C6—C7—C9	118.33 (13)	C12—C10—C11	113.15 (13)
C6—C7—C8	120.77 (14)	H12B—C12—H12C	109.5
O1—C9—O3	117.68 (13)	H12B—C12—H12A	109.5
O1—C9—C7	126.57 (14)	H12C—C12—H12A	109.5
O2—C8—O4	118.03 (13)	H11A—C11—H11C	109.5
O2—C8—C7	125.08 (14)	H11A—C11—H11B	109.5
N2—C6—H6	117.3	H11C—C11—H11B	109.5
N2—C6—C7	125.42 (14)	C2—C1—H1	118.2
C7—C6—H6	117.3	C2—C3—H3	120.6
N1—C1—H1	118.2	C2—C3—C4	118.84 (15)
N1—C1—C2	123.64 (15)	C10—C12—H12B	109.5
N1—C5—N2	116.58 (13)	C10—C12—H12C	109.5
N1—C5—C4	124.30 (14)	C10—C12—H12A	109.5
C1—C2—H2A	120.5	C10—C11—H11A	109.5
C9—O3—C10	118.08 (11)	C10—C11—H11C	109.5
C9—C7—C8	120.72 (13)	C10—C11—H11B	109.5
C8—O4—C10	117.75 (11)		
			
C6—N2—C5—N1	−4.6 (2)	C9—O3—C10—C11	−165.07 (13)
C6—N2—C5—C4	175.96 (15)	C9—C7—C8—O4	−9.6 (2)
C6—C7—C9—O3	−177.16 (12)	C9—C7—C8—O2	168.03 (15)
C6—C7—C9—O1	6.0 (2)	C8—O4—C10—O3	47.99 (17)
C6—C7—C8—O4	175.36 (13)	C8—O4—C10—C12	−74.10 (16)
C6—C7—C8—O2	−7.0 (2)	C8—O4—C10—C11	162.95 (12)
N2—C6—C7—C9	−177.35 (14)	C8—C7—C9—O3	7.7 (2)
N2—C6—C7—C8	−2.2 (2)	C8—C7—C9—O1	−169.19 (15)
N2—C5—C4—C3	179.53 (14)	C5—N1—C1—C2	0.7 (2)
C7—C6—N2—C5	−178.57 (14)	C4—C3—C2—C1	−0.7 (3)
N1—C1—C2—C3	0.0 (3)	C2—C3—C4—C5	0.6 (2)
N1—C5—C4—C3	0.2 (2)	C10—O3—C9—O1	−159.37 (14)
C1—N1—C5—N2	179.83 (13)	C10—O3—C9—C7	23.48 (19)
C1—N1—C5—C4	−0.8 (2)	C10—O4—C8—O2	162.84 (14)
C9—O3—C10—O4	−50.35 (17)	C10—O4—C8—C7	−19.38 (19)
C9—O3—C10—C12	71.76 (16)		
